# Downregulation of CD45 Signaling in COVID-19 Patients Is Reversed by C24D, a Novel CD45 Targeting Peptide

**DOI:** 10.3389/fmed.2021.675963

**Published:** 2021-08-03

**Authors:** Danny Alon, Yossi Paitan, Eyal Robinson, Nirit Ganor, Julia Lipovetsky, Rinat Yerushalmi, Cyrille J. Cohen, Annat Raiter

**Affiliations:** ^1^Department of Medicine A, Meir Medical Center, Kfar Saba, Israel; ^2^Sackler School of Medicine, Tel Aviv University, Tel Aviv, Israel; ^3^Microbiology Laboratory, Meir Medical Center, Kfar Saba, Israel; ^4^Department of Medicine B, Meir Medical Center, Kfar Saba, Israel; ^5^Felsenstein Medical Research Center, Rabin Medical Center, Petach Tikva, Israel; ^6^Laboratory of Tumor Immunotherapy, The Goodman Faculty of Life Sciences, Bar Ilan University, Ramat Gan, Israel

**Keywords:** CD45, COVID-19, PBMC, Src family of tyrosine kinases, immunosuppression

## Abstract

CD45, the predominant transmembrane tyrosine phosphatase in leukocytes, is required for the efficient induction of T cell receptor signaling and activation. We recently reported that the CD45-intracellular signals in peripheral blood mononuclear cells (PBMCs) of triple negative breast cancer (TNBC) patients are inhibited. We also reported that C24D, an immune modulating therapeutic peptide, binds to CD45 on immune-suppressed cells and resets the functionality of the immune system via the CD45 signaling pathway. Various studies have demonstrated that also viruses can interfere with the functions of CD45 and that patients with severe acute respiratory syndrome coronavirus-2 (SARS-CoV-2) are immune-suppressed. Given the similarity between the role of CD45 in viral immune suppression and our findings on TNBC, we hypothesized that the C24D peptide may have a similar “immune-resetting” effect on PBMCs from COVID-19 patients as it did on PBMCs from TNBC patients. We tested this hypothesis by comparing the CD45/TCR intracellular signaling in PBMCs from ten COVID-19 patients vs. PBMCs from ten healthy volunteers. Herein, we report our findings, demonstrating the immune reactivating effect of C24D via the phosphorylation of the tyrosine 505 and 394 in Lck, the tyrosine 493 in ZAP-70 and the tyrosine 172 in VAV-1 proteins in the CD45 signaling pathway. Despite the relatively small number of patients in this report, the results demonstrate that C24D rescued CD45 signaling. Given the central role played by CD45 in the immune system, we suggest CD45 as a potential therapeutic target.

## Introduction

CD45 is a transmembrane protein tyrosine phosphatase receptor type C (PTPRC), expressed exclusively in leukocytes, with double opposing effects on T cell receptor (TCR) activity (1, 2). On the one hand, CD45 plays an inhibitory function involving the dephosphorylation of the tyrosine 394 (Y394) in the lymphocyte-specific protein tyrosine kinase (Lck), preventing its activation. On the other hand, CD45 plays the role of an activator when it dephosphorylates the tyrosine 505 (Y505), an inhibitory site at the C-terminal end of the non-receptor tyrosine-Src kinases. Activated Lck phosphorylates the immunoreceptor tyrosine-based activation motifs (ITAMs) of the T cell receptor (TCR)/CD3 complex. The phosphorylated ITAMs recruit the Zeta-chain-associated protein kinase 70 (ZAP-70), via its Src homology 2 (SH2) domains. Finally, for TCR activation, CD3-bound ZAP-70 is activated by both Lck and (trans)-auto-phosphorylation at the ZAP70 tyrosine 493 (Y493) (3–5). The ZAP70 tyrosine kinase transmits a downstream signal leading to VAV-1 phosphorylation and activation (6, 7).

We recently reported an immune escape mechanism in TNBC patients showing that CD45's intracellular signals are inhibited (8). We also reported that C24D, a previously described immune modulating therapeutic peptide (9), binds to the CD45 receptor of the TNBC-suppressed immune cells and reverses immune-suppression, via the CD45 signaling pathway (8). C24D-binding to CD45 in the immune-suppressed cells resulted in immune reactivation and specific tumor killing.

Various studies have demonstrated that also viruses can interfere with the functions of CD45 (10). The underlying mechanism of the viral/CD45 immune-suppressive interaction was elucidated on cytomegaloviruses, adenoviruses and others (11, 12). The protein UL11 from the cytomegalovirus (CMV) and the protein E3/49K from adenovirus (AdV) are known to bind to CD45 (11, 12). The sec49K viral protein, derived from E3/49K, was found to affect CD45 in non-infected adjacent and distant cells (13). Additionally, functional studies showed that sec49K can suppress the activation, signaling, cytotoxicity and cytokine production of T and NK cells (13).

Coronavirus disease 2019 (COVID-19), first identified in December 2019 in Wuhan, China, is an infectious disease caused by severe acute respiratory syndrome coronavirus 2 (SARS-CoV-2). By March 2021, the number of COVID-19 confirmed cases globally was 113,467,303 and 2,520,550 deaths were reported (14, 15).

It has been shown that patients with severe acute respiratory syndrome coronavirus-2 (SARS-CoV-2) are immune-suppressed (16–18). Given the similarity between the CD45 viral immune suppression reported by others and our oncology findings, we hypothesized that the C24D peptide may have a similar immune-resetting effect on PBMCs from COVID-19 patients as it did on PBMCs from TNBC patients. We demonstrate in this Brief Report the effect of adding C24D to PBMCs obtained from ten hospitalized COVID-19 patients on the phosphorylation of Lck, ZAP-70 and VAV-1 proteins in the CD45 signaling pathway.

## Methods

### C24D Peptide Synthesis

The C24D peptide is a 25 amino acid homodimer peptide with a disulphyde bond at CG (CGHHLLRPRRRKRPHSIPTPILIFRSP), synthesized by Synpeptide Co., Ltd. (Shanghai, China). HPLC showed purity >97%.

### PBMC Isolation

PBMC was isolated from blood samples of healthy female donors and hospitalized COVID-19 patients and was obtained from the Blood Bank Mada Tel HaShomer and Meir Medical Center, respectively. The protocol was approved by the Institutional Review Board at Meir Medical Center, Israel (0094-20-MMC). Samples were isolated by Ficoll–Hypaque density gradient (*d* = 1,077 g/mL, Ficoll-Paque Plus, GE Healthcare, Upsalla, Sweden) by centrifugation at 650 × g for 30 min.

### Patients and Data Collection

We conducted analyses of ten COVID-19 patients hospitalized in the Meir Medical Center, Kfar Saba, Israel. COVID-19 was diagnosed by RT-PCR, based on criteria issued by the National Health Commission of Israel. Only patients with a positive, laboratory-confirmed test for SARS-CoV-2 and who suffered from sufficiently serious COVID-19 symptoms to warrant hospitalization were included. Baseline and follow-up data for all patients was obtained from the electronic medical record system. All ten patients were still hospitalized at the time of blood extraction; five patients already had a negative confirmed RT-PCR test for SARS-CoV-2 at the time of blood extraction and five still had a positive result. The Ethics Committees of the Meir Medical Center and the Israel Ministry of Health approved the study and written informed consent was obtained from all subjects. All ten patients were categorized as non-severe cases.

### CD45 Signal Transduction

C24D, at 10 μg/ml, was added immediately after PBMC isolation and incubated for 5, 15, 30, 60 min and 24 h at room temperature. PBMCs were centrifuged and re-suspended in 0.12 ml of lysis buffer (20 mM Tris-HCl pH 7.5, 150 mM NaCl, 1 mM NaF, 2 mM Na_3_VO_4_, 1% NP40, 10 mM b-glycerophosphate, 30% glycerol, 1 mM EDTA, 0.5% sodium-deoxycholate, 0.5% protease inhibitor cocktail), followed by one freeze-thaw cycle of 20 min. Cells were harvested and centrifuged (14,000 rpm, 15 min, 4°C). The supernatants were collected, and aliquots were separated on 10% SDS PAGE, followed by Western blotting with anti-phospho-Lck Y505 (0.5 μg/ml, ab4901, Abcam, Cambridge, UK), anti-phospho-Lck Y394 (0.25 μg/ml, ab201567, Abcam), anti phospho-VAV-1 Y174 (0.23 μg/ml, ab76225, Abcam) and anti-phospho-ZAP70 Y493 (1 μg/ml, ab194800, Abcam). GAPDH (1 μg/ml, ab9485, Abcam) was added as a control for sample loading. After several washings, the secondary antibody, IRDye 800CW Goat anti-Rabbit or IRDye 680CW Goat anti mouse (1 μg/ml, LI-COR, Nebraska, USA) was added for 1 h.

### Quantification Methods

The membrane was analyzed by Odyssey 2.1 (Infrared Imaging System) for specific band identification. Quantification of phosphorylation was done by Image J (NIH, USA). Percentage (%) of maximal phosphorylation of phosphorylated proteins were first normalized to the levels obtained with GAPDH, respectively, and the activation values were normalized for each time point vs. its control, without C24D (e.g., C24D + lymphocytes vs. lymphocytes control). The values obtained were then expressed as % of maximal activation that was observed in each experiment, at each time point. All the results were normalized with GAPDH as the reference protein.

### Statistics

Data to compare results between the patients and healthy groups we used the independent (two-tailed) *t*-test. For multiple comparisons we used the One-Way ANOVA test. Significance was defined as *p* < 0.05.

## Results

### No Correlation Between Clinical Characteristics of Patients and RT-PCR Results Was Found

Baseline clinical data and laboratory findings are shown in Table 1. The median age of the study population was 60.7 (age range, 42–79) years, 50% were female. Blood from five patients was obtained toward the end of hospitalization (RT-PCR negative) and from five patients shortly after being hospitalized (RT-PCR positive). All patients were hospitalized for a minimum of 1 week (range, 7–45 days). Main risk factors for severe Covid-19 disease in our patient population included obesity (four patients) and impaired glucose tolerance (three patients).

**Table 1:** Clinical characteristics of the ten hospitalized Covid-19 patients.

**Patient**	**Age**	**Gender**	**PCR**	**Weight**	**WBC × 10^**3**^**	**RBC × 10^**6**^**	**Hb**	**Glucose**	**Ferritin**	**Secondary infections**	**Days of hospitalization**
1	78	M	Neg	76	7.14	4.04	12.2	107	760	None	45
2	79	F	Neg	54	11.99	4.29	12.6	N/A	N/A	None	30
3	55	M	neg	103	89.2	5.36	13.3	207	405	None	11
4	42	F	Pos	115	5.2	5.01	13.7	90	833	None	19
5	46	M	Pos	110	4.74	4.91	14.2	96	597	None	11
6	70	M	Neg	75	6.02	5.34	15.8	97	690	None	14
7	74	M	Pos	64	5.84	4.7	14.3	80	681	Klebsiella-colonization	16
8	68	F	Neg	n/a	9.8	5.27	12.2	119	147	Serratia bacteremia	12
9	42	F	Pos	63	7.71	4.11	12.3	94	N/A	None	8
10	53	F	Pos	n/a	4.57	4.46	12.6	96	398	None	8

*WBC, White blood cell count; RBC, red blood cell count; Hb, Hemoglobin; PCR, RT PCR results for Covid-19 disease*.

### Addition of C24D to PBMCs From COVID-19 Patients Resets the Phosphorylation of Src Protein Kinases

**Figure 1:**
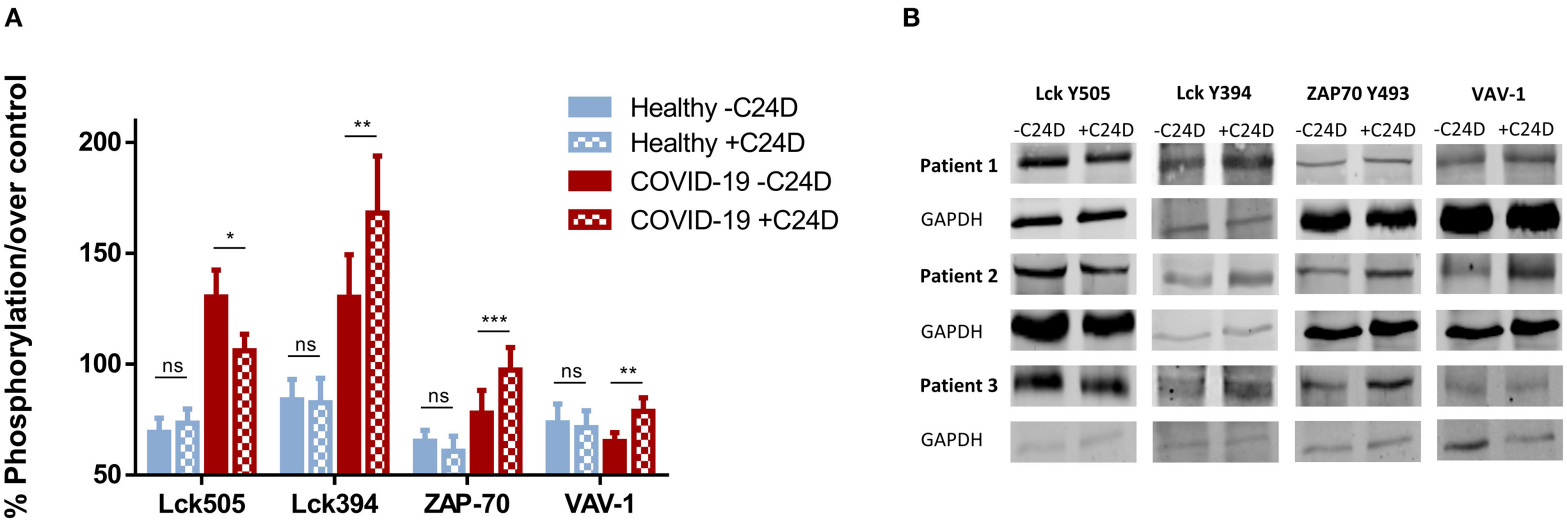
C24D binding to CD45 triggers the CD45 signaling pathway: PBMCs from ten hospitalized patients, ± C24D treatment, were lysed, separated on 10% SDS and blotted with antibodies for the determination of phosphorylation of the tyrosine 505 and 394 in Lck, the tyrosine 493 in ZAP-70 and the tyrosine 174 in VAV-1. PBMCs from healthy volunteers served as controls. The percentage of phosphorylation was calculated individually vs. GAPDH as protein control. **(A)** Percentage change of protein phosphorylation in PBMCs from 10 patients, compared to 10 healthy donors, after addition of C24D for 5–60 min to patient PBMCs, or 24 h to PBMCs from healthy donor (values represent mean± SE, *p < 0.01, **p < 0.005, ***p < 0.0001). **(B)** Western blot results obtained in three representative patients for each phosphorylated protein, ± C24D.

Using western blot analysis, we determined the phosphorylation of proteins in the CD45 signaling pathway resulting from the addition of the C24D peptide to fresh PBMCs from ten COVID-19 patients. The results were compared to the same measurements using PBMCs from healthy volunteers (Figure 1). Addition of C24D for 5–60 min to fresh PBMCs from the COVID-19 patients resulted in activation of Lck (a member of the Src protein kinases).

Statistically significant differences were found between four groups: (1) patients, (2) healthy volunteers, (3) before and (4) after the addition of C24D to PBMCs, (*p* < 6.8 × 10^−5^, *p* < 0.02, *p* < 0.017, and *p* < 0.05) in Lck Y505, Lck Y394, ZAP-70 and VAV-1 phosphorylation, respectively, as determined by One-Way ANOVA. The statistically significant results correspond to the effect of the addition of C24D to PBMCs from COVID-19 patients.

As depicted in Figure 1A, a significant decrease in the phosphorylation of the inhibitory tyrosine 505 in Lck was observed (from 130.35 ± 12.08% to 103.12 ± 7.35%, *p* < 0.01) only in patients. In parallel, we observed a significant increase in the phosphorylation of the tyrosine 394 of Lck (from 130.19 ± 19.23% to 168.25 ± 25.69%, *p* < 0.007). Consequently, ZAP-70 was activated, as evidenced by a significant increase in the phosphorylation of the tyrosine 493 in ZAP-70 (from 77.97 ± 10.17% to 97.34 ± 10.14%, *p* < 0.0001). A similar pattern was observed for VAV-1 phosphorylation. Addition of C24D to fresh PBMCs from COVID-19 patients significantly increased VAV-1 phosphorylation (from 65.20 ± 3.99% to 78.73 ± 6.09%, *p* < 0.005, Figure 1A). In contrast to the C24D-induced immune reactivation observed in PBMCs from COVID-19 patients, no significant effect was seen on Lck (Y505, *p* < 0.122; Y394, *p* < 0.301), ZAP70 (*p* < 0.08) and VAV-1 (*p* < 0.274) phosphorylation when fresh PBMCs from healthy donors were incubated with C24D for 5 min to 24 h (Figure 1A). Figure 1B depicts the western blot results obtained from three representative Covid-19 patients. Supplementary Figure 1 depicts the remaining 7 COVID-19 patients' western blot results.

**Figure 2:**
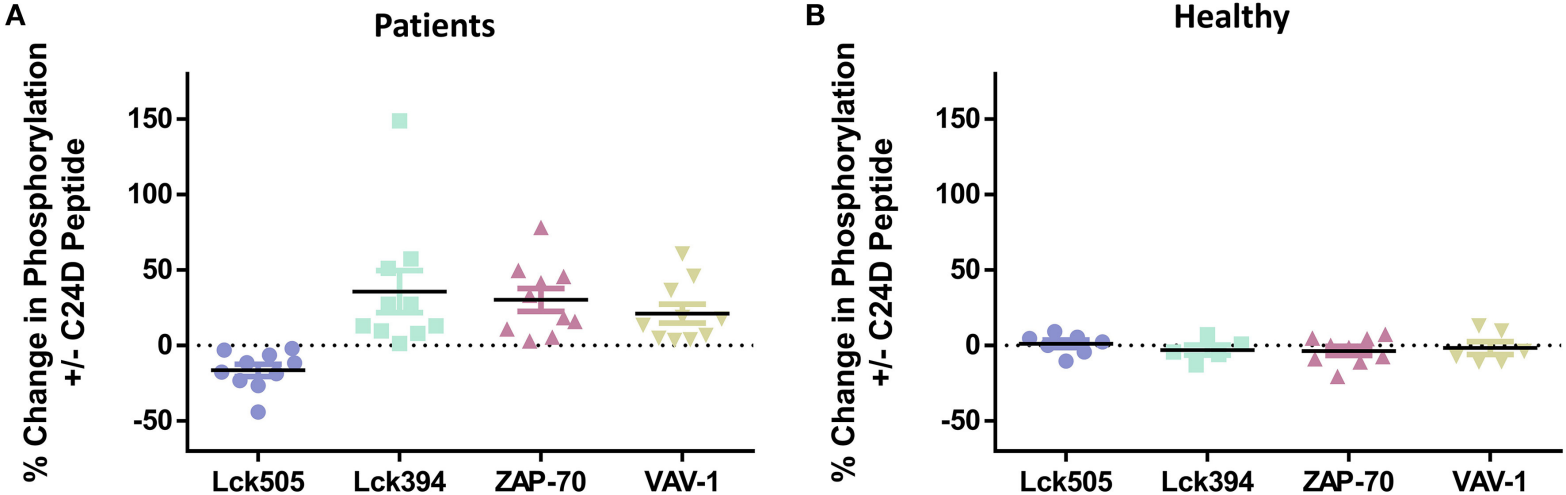
C24D reversed CD45 immune suppression signaling only in patients: **(A)** Percentage change of Lck, ZAP-70 and VAV-1 phosphorylation in PBMCs from each of the 10 patients, after addition of C24D for 5–60 min, normalized to control without C24D. **(B)** Percentage change of Lck, ZAP-70 and VAV-1 phosphorylation in PBMCs from healthy donors, after addition of C24D for at least 24 h, normalized to control without C24D.

To better understand the effect of the C2D peptide on the CD45 signaling pathway, we analyzed the percent change in phosphorylation for each of the 4 relevant proteins, for every PBMC sample from the ten COVID-19 patients, +C24D vs. –C24D (Figure 2A). The average change resulting from treatment with the C24D peptide on the phosphorylation of the inhibitory Lck tyrosine 505 and the immune-stimulating tyrosine 394 was −18.6 and +29.2%, respectively. ZAP-70 phosphorylation increased by an average of 24.8% and VAV-1 phosphorylation by 20.7%. Treatment of PBMCs from healthy volunteers with C24D did not cause a significant change in the phosphorylation of the four relevant proteins (Figure 2B). Thus, in this study, C24D specifically altered the phosphorylation pattern of key CD45-signaling molecules and did so only in PBMCs derived from COVID-19 patients.

Supplementary Figure 2 shows the pattern of phosphorylation of Lck, ZAP-70 and VAV-1 over time (from 5 to 30 min), obtained by western blot analysis, for each COVID-19 patient. Lck 505 de-phosphorylation and Lck 394 phosphorylation are completed 30 min after addition of C24D, to PBMCs of all patients. Five minutes and 15 min after treatment, ZAP-70 and VAV-1 are phosphorylated, respectively, in all tested PBMCs.

### No Correlation Was Observed Between Clinical Characteristics and the Protein Phosphorylation Pattern

No correlation was observed between the clinical characteristics (age, weight, glucose and ferritin levels) of the ten hospitalized COVID-19 patients and the percentage of Lck505, Lck394, ZAP70, and VAV-1 phosphorylation induced by C24D, as determined by One-Way ANOVA test.

Interestingly, there was no difference in the C24D-induced CD45 signaling between the five hospitalized patients who already had negative RT-PCR results and the 5 hospitalized patients who still had positive RT-PCR results, implying that the patients whose RT-PCR results reverted to negative were still immunosuppressed.

**Figure 3:**
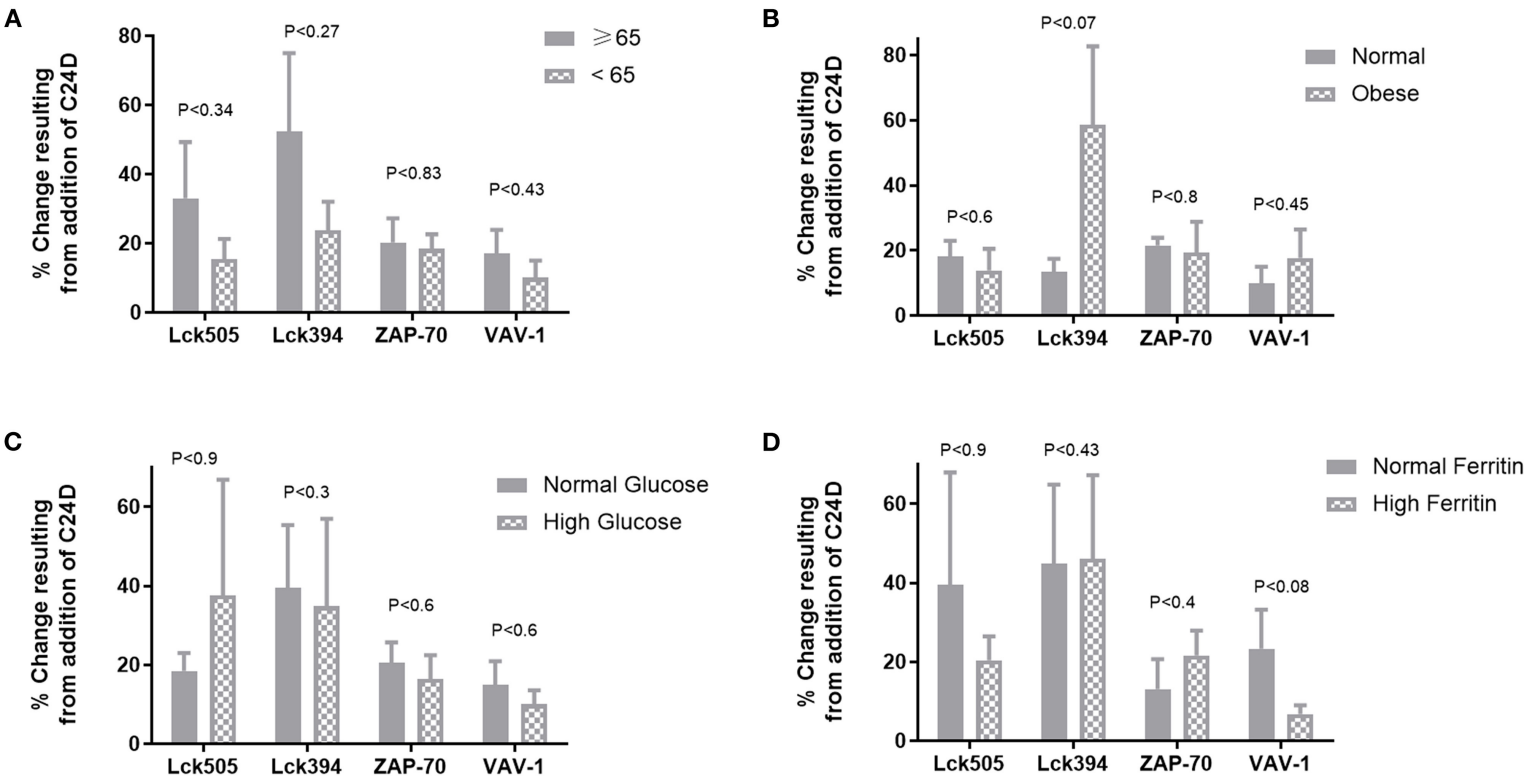
Correlations between clinical characteristics and the protein phosphorylation pattern: no significant differences were found between age, weight, glucose levels or ferritin values and the change of phosphorylation in proteins, normalized to control without C24D. **(A)** Age, in two groups ≥65 vs. <65 years of age, *p* < 5.7 × 10^−5.^ **(B)** Weight, in two groups, normal weight: 66.4 ± 9.1 vs. obese: 109.3 ± 6.02, *p* < 0.00004 **(C)** Glucose, in two groups, normal glucose: 91.9 ± 0.02 vs. high glucose: 144.3 ± 54, *P* < 0.027 **(D)** Ferritin, in two groups, normal ferritin: 316 ± 145 vs. high ferritin: 712 ± 79, *p* < 0.0028.

We divided the patients into two significantly different age groups: ≥65 and <65 (*p* < 5.73E-05, Figure 3A). No correlation between the response to C24D in Lck, ZAP-70, and VAV-1 activation to the age of patients was found.

In spite of the small number of patients in each of the weight, glucose and ferritin values sub-groups, we nonetheless evaluated the effect of C24D on CD45 signaling. We found that the difference between the two weight sub-groups (obese vs. normal) was statistically significant (*p* < 0.025). However, no correlation between the phosphorylation of Lck505, Lck394, ZAP70, and VAV-1 and weight was observed (Figure 3B). Addition of C24D induced Lck, ZAP70 and VAV-1 activation equally in both sub-groups. A similar pattern of protein phosphorylation was observed in patients with normal and high glucose levels (*p* < 0.0004, Figure 3C) and in patients with normal and high ferritin values (*p* < 0.003, Figure 3D).

## Discussion

It has been reported that patients infected with the SARS-CoV-2 are immune-suppressed (16–20). In some studies, immunosuppression was described as a consequence of a drastic reduction in the number of both CD4+ and CD8+ T cells in moderate and severe COVID-19 patients (21). This is consistent with reports that viruses developed immune evasion strategies similar to those deployed by tumors (22, 23).

In this short report, we demonstrated that treatment of PBMCs from ten hospitalized COVID-19 patients with C24D resulted in the reactivation of CD45 key-signaling molecules: Lck, ZAP-70 and VAV-1. Binding of C24D to the CD45 receptor provoked a decrease in the phosphorylation of the inhibitory tyrosine 505 and an increase in the phosphorylation of the tyrosine 394 in Lck, inducing its activation. The tyrosine 493 in ZAP70 and tyrosine 174 in VAV-1 were phosphorylated, resulting in TCR activation (24, 25).

Given the pivotal role of CD45 in the immune system (26–28), it is not surprising that viruses interfere with the activity of CD45 to dampen the immune response.

Similar to our findings on TNBC tumors (8), viral interference with the functions of the receptor tyrosine phosphatase CD45 have been widely reported. It was demonstrated that CD45 functions are crucial for stimulating a protective immune response against Herpes simplex virus type 1 (HSV-1) (29). When CD45 is down-regulated, the immune system fails to control HSV-1 infection and to prevent HSV-1 associated encephalitis. In adenovirus, the secreted protein sec49K derived from the viral protein E3/49K was found to bind to CD45 receptor resulting in a significant decrease in the activation of Src tyrosine proteins kinases and ZAP-70, causing suppression of activation of T and NK cells (13).

In this study, we found that on binding to CD45's extracellular domain of PBMCs of COVID-19 patients, C24D reverses the deactivation of kinases involved in CD45/TCR signaling. Conversely, in PBMCs from healthy volunteers, C24D did not change the CD45 signaling pattern, suggesting that C24D acts only on immune-suppressed cells. The focus on T cell re-activation originated from reported studies which demonstrated that the severity of COVID-19 inversely correlates with T-cell immunity of the host. Although T cells cannot prevent infections, in COVID-19 patients, killer T cells mean the difference between a mild infection and a severe one (30, 31).

The five hospitalized COVID-19 patients who, at the time blood was drawn, already had negative RT-PCR results, presented the same response to C24D treatment as did the five RT-PCR positive Covid-19 patients. This suggests that some immunosuppression may endure for some time after the elimination of the SARS-CoV-2 virus.

Age-associated alterations in the immune system contribute to the increased incidence and severity of infectious diseases in elderly patients (32). There is a consensus that the elderly (≥65 age) is the population group most vulnerable to COVID-19. We found no statistically significant difference in the effect of C24D on CD45 signaling in COVID-19 patients under 65 and over 65 years of age, suggesting that C24D might be effective also in patients ≥65 years of age.

In each of the weight, ferritin and glucose values sub-groups, no statistically significant differences in the phosphorylation of Lck505, Lck394, ZAP70, and VAV-1 were observed. Ferritin values aroused our attention due to C24D being a peptide derived from the placental immunomodulatory ferritin (9).

Due to the small cohort of patients, it is possible that a correlation between the effect of treatment with the C24D peptide on CD45 downstream signaling and patients' clinical characteristics was masked.

Other report limitation was related to the hospital ethics committee that did not allow us to perform experiments with serum or plasma from COVID-19 patients due to standard biosecurity and institutional safety. The quantity of PBMCs recovered from each patient was minimal and lyzed for virus neutralization. The amount of cells only sufficed for the study of protein phosphorylation and not for functional assays.

COVID-19 is a multifaceted illness that affects different people in different ways (33). The long-term effectiveness of COVID-19 vaccines is yet to be determined and may be vulnerable to virus mutations (33). Thus, there is a need to find additional therapeutic strategies to tackle COVID-19.

In spite of the relatively small number of patients in this report, the results showed that C24D rescued CD45 signaling. T cell re-activation through the CD45 molecular pathway is potentially a new therapeutic strategy against immunosuppression induced by coronavirus. Given that some cases of immune overactivation (cytokine storm) have been reported (34, 35), a molecule such as C24D, which does not activate, but rather resets the immune system, could likely serve as an important therapeutic option in the treatment of the current, and possibly future generations of SARS-CoV-2.

## Data Availability Statement

The raw data supporting the conclusions of this article will be made available by the authors, without undue reservation.

## Ethics Statement

The studies involving human participants were reviewed and approved by Meir Medical Center and Israel Ministry of Health. The patients/participants provided their written informed consent to participate in this study.

## Author Contributions

DA performed and managed the ethical approval for patient recruitment and sample collection. YP analyzed and contributed to data analysis and visualization. ER contributed to recruitment, sample collection and data analysis, and visualization. NG and JL performed the experiments described in this manuscript in patients and healthy volunteers' samples. RY and CC revised and critically edited the manuscript. AR wrote, reviewed and edited the manuscript, and analyzed the data. All authors contributed to the article and approved the submitted version.

## Conflict of Interest

The authors declare that the research was conducted in the absence of any commercial or financial relationships that could be construed as a potential conflict of interest. The reviewer OZ declared a past collaboration with several of the authors JL, AR, and RY to the handling editor.

## Publisher's Note

All claims expressed in this article are solely those of the authors and do not necessarily represent those of their affiliated organizations, or those of the publisher, the editors and the reviewers. Any product that may be evaluated in this article, or claim that may be made by its manufacturer, is not guaranteed or endorsed by the publisher.
