# Supplementary figures and images for: Downregulation of CD45 Signaling in COVID-19 Patients Is Reversed by C24D, a Novel CD45 Targeting Peptide
Source: Front Med (Lausanne). 2021 Aug 3;8:675963. doi: 10.3389/fmed.2021.675963 (PMC8369232; doi:10.3389/fmed.2021.675963)

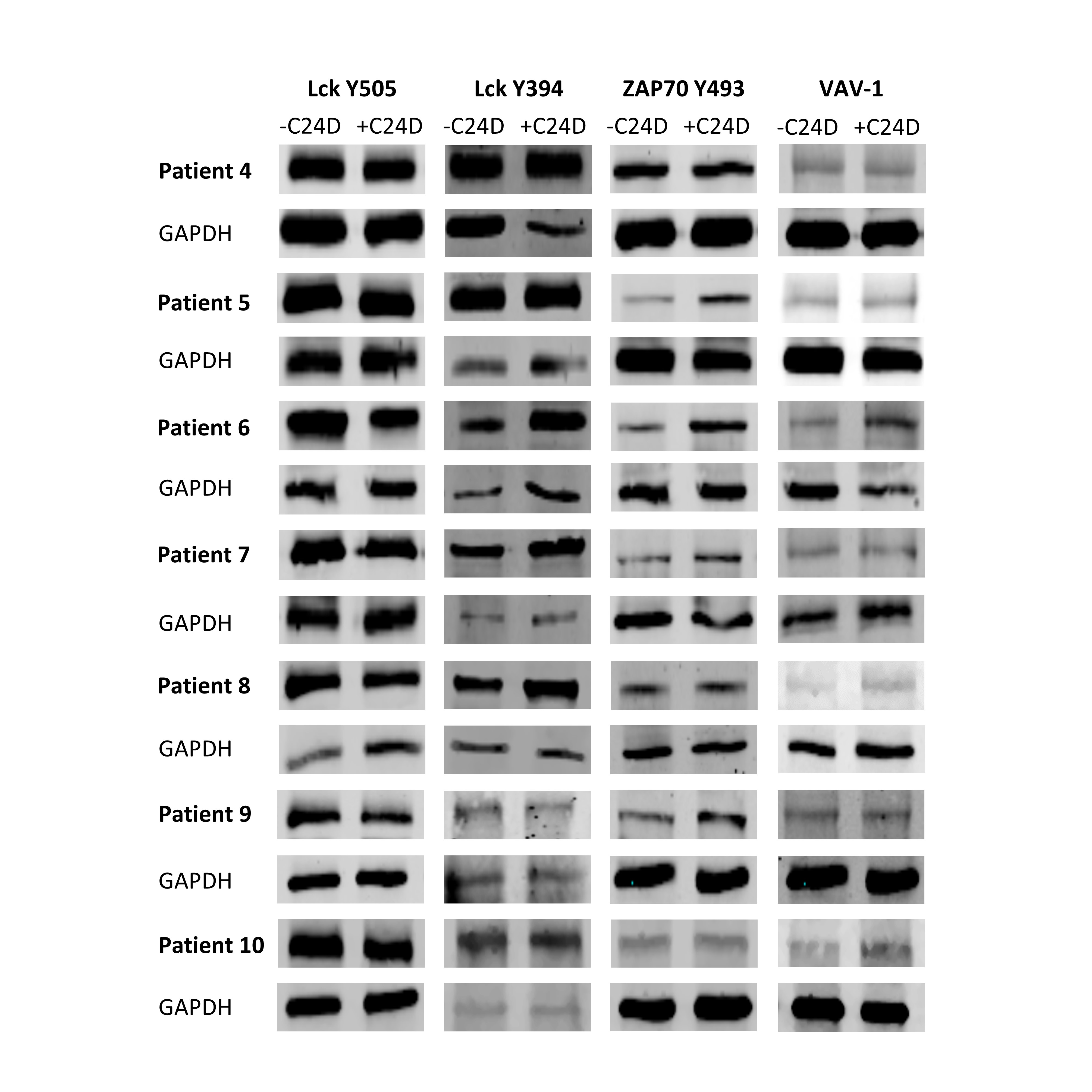

Supplement: Supplementary file 1 [file Image_1.JPEG]

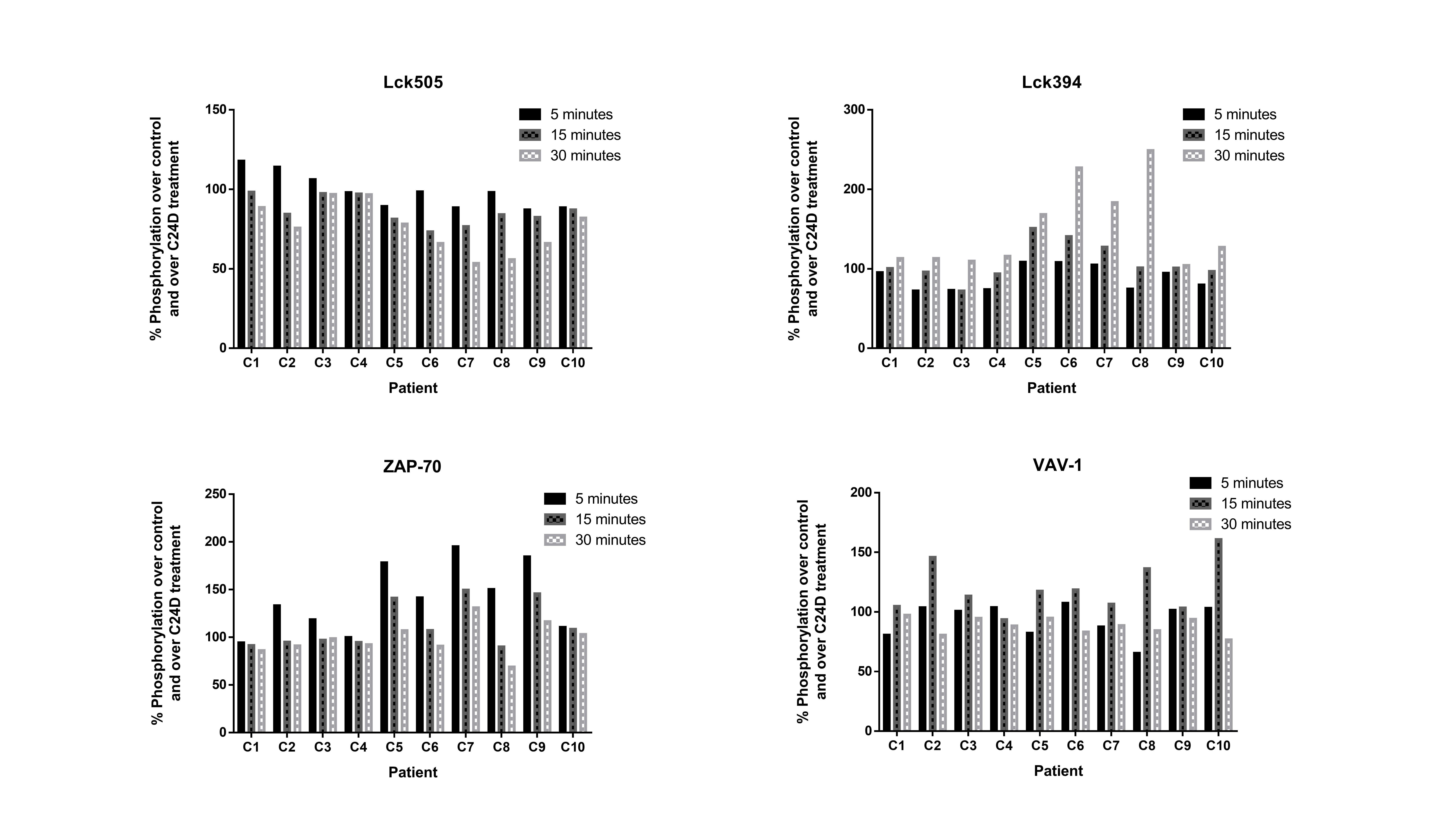

Supplement: Supplementary file 2 [file Image_2.JPEG]
